# Subtypes of primary colorectal tumors correlate with response to targeted treatment in colorectal cell lines

**DOI:** 10.1186/1755-8794-5-66

**Published:** 2012-12-31

**Authors:** Andreas Schlicker, Garry Beran, Christine M Chresta, Gael McWalter, Alison Pritchard, Susie Weston, Sarah Runswick, Sara Davenport, Kerry Heathcote, Denis Alferez Castro, George Orphanides, Tim French, Lodewyk FA Wessels

**Affiliations:** 1 Division of Molecular Carcinogenesis, The Netherlands Cancer Institute, Amsterdam, The Netherlands; 2 Faculty of EEMCS, Delft University of Technology, Delft, The Netherlands; 3 Oncology iMed, AstraZeneca R&D, Alderley Park, Macclesfield, United Kingdom; 4 Personalised Healthcare and Biomarkers Group, AstraZeneca R&D, Alderley Park, Macclesfield, United Kingdom; 5 Department of Molecular Carcinogenesis, Plesmanlaan 121, Amsterdam, 1066CX, The Netherlands

**Keywords:** Colorectal cancer, Tumor subtyping, Cell lines, Targeted therapy

## Abstract

**Background:**

Colorectal cancer (CRC) is a heterogeneous and biologically poorly understood disease. To tailor CRC treatment, it is essential to first model this heterogeneity by defining subtypes of patients with homogeneous biological and clinical characteristics and second match these subtypes to cell lines for which extensive pharmacological data is available, thus linking targeted therapies to patients most likely to respond to treatment.

**Methods:**

We applied a new unsupervised, iterative approach to stratify CRC tumor samples into subtypes based on genome-wide mRNA expression data. By applying this stratification to several CRC cell line panels and integrating pharmacological response data, we generated hypotheses regarding the targeted treatment of different subtypes.

**Results:**

In agreement with earlier studies, the two dominant CRC subtypes are highly correlated with a gene expression signature of epithelial-mesenchymal-transition (EMT). Notably, further dividing these two subtypes using iNMF (iterative Non-negative Matrix Factorization) revealed five subtypes that exhibit activation of specific signaling pathways, and show significant differences in clinical and molecular characteristics. Importantly, we were able to validate the stratification on independent, published datasets comprising over 1600 samples. Application of this stratification to four CRC cell line panels comprising 74 different cell lines, showed that the tumor subtypes are well represented in available CRC cell line panels. Pharmacological response data for targeted inhibitors of SRC, WNT, GSK3b, aurora kinase, PI3 kinase, and mTOR, showed significant differences in sensitivity across cell lines assigned to different subtypes. Importantly, some of these differences in sensitivity were in concordance with high expression of the targets or activation of the corresponding pathways in primary tumor samples of the same subtype.

**Conclusions:**

The stratification presented here is robust, captures important features of CRC, and offers valuable insight into functional differences between CRC subtypes. By matching the identified subtypes to cell line panels that have been pharmacologically characterized, it opens up new possibilities for the development and application of targeted therapies for defined CRC patient sub-populations.

## Background

Colorectal cancer (CRC) is the third most common cancer, with an estimated 1.2 million cases and 608,700 deaths worldwide in 2008 [1]. While the overall effects and interactions of environmental and lifestyle factors [2], and inherited and acquired genetic and epigenetic alterations [3-5] on CRC development are still incompletely understood, knowledge has improved in recent years.

The generally assumed model of CRC development implies a sequence of events leading from adenoma formation to carcinoma that is caused and accompanied by genetic and epigenetic events [3]. Different molecular phenotypes have been used to define CRC subtypes [6,7], for instance microsatellite instability (MSI) [8], epigenetic alterations, such as the methylation state of CpG islands [9], the location of the tumor in the colon/rectum, and mutations in genes, such as KRAS or BRAF. Key pathways that have been implicated in CRC include Wnt/ß-catenin, TGF-ß, MAPK, and PI3K signaling [3].

Intense research has been directed at the discovery of biomarkers that are predictive of disease progression or treatment response, albeit with limited success. For Stage II and III CRC, microsatellite instability was found to be predictive of better prognosis [6]. Tumor staging, microsatellite instability, and loss of heterozygosity on Chromosome 18q have been used as prognostic factors for treatment with chemotherapy [10]. Targeted monoclonal antibodies against VEGF-A and EGFR have been approved for therapy of advanced CRC. While resistance to EGFR antibodies is associated with mutations in the KRAS gene, an effect of BRAF mutations has not been proven conclusively [11]. Furthermore, a large proportion of patients with wild-type KRAS do not respond to EGFR inhibition [12,13]. On the other hand, inhibition of EGFR has recently been shown to have a synergistic effect on BRAF(V600E) inhibition [14]. In order to better select patients that will respond to targeted treatment Dry and colleagues employed a pathway-based approach to derive a gene expression signature predictive of sensitivity to MEK inhibition by assessing activation of MEK and compensatory signaling from other RAS effectors [15]. These efforts highlight the importance of gaining a better understanding of the molecular differences between CRC subtypes at the pathway level. Since clinical response data for targeted treatments are very limited, cell line models have become an increasingly important tool for research into the molecular basis of different cancers and linking molecular features to phenotypes such as drug response [16,17].

A number of studies have been conducted in CRC, often in a supervised fashion, to develop gene expression signatures capable of identifying patient populations at high risk of recurrence [18-26]. In other cases, authors developed signatures of differentially expressed genes that allow distinguishing between different tumor stages [27,28], or normal samples from tumors and metastases [29-31]. Recently, unsupervised analyses have been conducted with the goal of discovering CRC subtypes and explain functional differences [32-34]. First, Loboda *et al.* described two major CRC subtypes which were shown to correlate with a signature of epithelial-mesenchymal-transition [32]. Later, Oh and colleagues applied hierarchical clustering to a CRC patient cohort and identified a gene signature that was associated with survival and response to chemotherapy [33]. Perez-Villamil *et al.* found four CRC subtypes based on hierarchical clustering including a stromal subtype that was associated with poor survival [34].

In the present study, we set out to discover subtypes of primary CRC tumors with the aim to better characterize their functional differences on the pathway level. In contrast to previous studies, we employed a new iterative clustering method which allows us to detect expression patterns of varying strength. Instead of relying on highly variable probe sets, our method employs randomly selected probe set groups that cover a large portion of the expression data. As a result, our method is unbiased with respect to prior knowledge about certain genes or pathways. Furthermore, we provide the first alignment of pharmacologically characterized cell line panels to the discovered tumor subtypes. This enables us to assess how well primary tumor subtypes are covered by available cell line panels. To the best of our knowledge, we provide the first attempt at deriving hypotheses about response of individual subtypes to targeted treatment. First, we identified two subtypes showing strong association with an EMT phenotype and significant differences in survival times and microsatellite status. A subsequent second split of these two subtypes yielded five subtypes providing a more fine grained stratification. We demonstrate that these subtypes can be robustly reproduced on an independent set of over 1600 CRC tumor samples drawn from 15 previously published studies. More importantly, repeating the subtyping procedure on an independent dataset resulted in discovery of highly similar subtypes. By applying the subtyping to 74 different CRC cell lines, we show that all tumor subtypes are represented in the cell lines implying that the cell lines largely reflect the gene expression heterogeneity present in tumors. The integration of pharmacology data reveals that cell lines assigned to specific subtypes show exquisite sensitivity to targeted inhibitors. This provides evidence that the subtyping can be used for developing and selecting targeted treatments for specific subpopulations of CRC tumors.

## Methods

### Tumor and cell line datasets

#### CRC Tumor data

**Figure 1:**
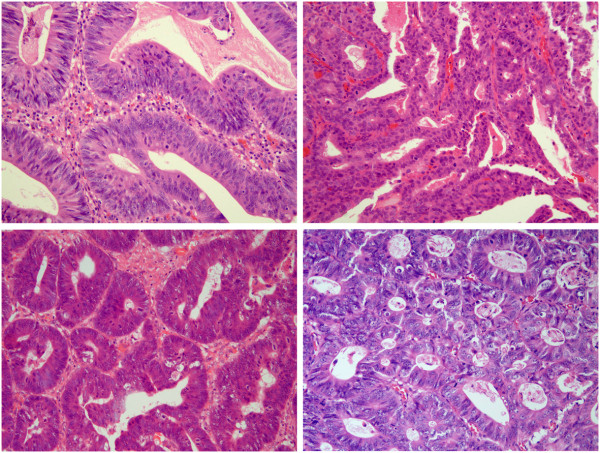
Histology images of four samples from AZTS (20x magnification).

We performed genome-wide mRNA expression profiling on 62 primary CRC samples (AZTS, GSE35896, Table 1, Figure 1) using Affymetrix HGU133plus2 GeneChips according to the manufacturer's protocol (Affymetrix, Santa Clara, CA). We also downloaded 15 CRC tumor sample expression datasets encompassing a total of 1643 samples from the Gene Expression Omnibus all hybridized on the same Affymetrix HGU133plus2 platform (see Additional file 1: Table S1).

**Table 1:** Characteristics of the tumors contained in set AZTS

**Variable**	**Value**	**Count**
Sex	Female/Male/unknown	30/32/1
Age	30 – 39	1
	40 – 49	4
	50 – 59	8
	60 – 69	25
	70 – 79	20
	80 – 89	4
AJCC stage	I/II/III/IV/unknown	0/26/7/1/28
TNM stages	T0/T1/T2/T4/TX	0/0/1/30/6/25
	N0/N1/N2/NX	8/7/3/44
	M0/M1/MX	49/1/12
Microsatellite instability status	MSI/MSS/unknown	5/56/1

#### CRC cell line data

We analyzed an available dataset consisting of gene expression profiles of 54 CRC cell lines (AZCL, Chresta CM *et al.*, in preparation) as well as MSI status and mutation status for KRAS, BRAF, p53, PiK3CA, APC, and PTEN. We downloaded one cell line gene expression dataset from the Gene Expression Omnibus (GSE8332 [35]) and data from the GSK Cancer Cell Line panel from caBIG at the National Cancer Institute (GSK, [36]). All three cell line panels were hybridized on the Affymetrix HGU133plus2 platform (see Additional file 1: Table S2). From ArrayExpress, we also downloaded a dataset containing the gene expression data for 34 large intestine cell lines hybridized on the Affymetrix HGU133A array (accession number E-MTAB-783 [16]). This dataset was generated in the Cancer Genome Project at the Wellcome Trust Sanger Institute and will be referred to as the ‘Sanger set’.

### Data analysis

We used R/Bioconductor software [37] for all processing of the microarray data prior to analysis. We normalized raw intensity values for each dataset independently using RMA as implemented in the *affy* package [38]. We mean-centered expression values for individual probe sets for determining differential expression, hierarchical clustering, and plotting heatmaps. We utilized 1 – Pearson correlation as distance measure and complete linkage for hierarchically clustering expression data. To perform the combined analysis of the sets AZCL, GSK, and GSE8332, we normalized gene expression data for these datasets together but mean-centered each set separately to subtract any batch effect. Data for the cell lines C10, C125PM, C80, C99, CCK81, DLD1, HCA46, HRA19, LS513, NCI747, Vaco10MS, Vaco4A, Vaco4S, and Vaco5 in the AZCL set were treated as separate batch for mean-centering. We averaged expression values from cell line replicates within each panel before assigning them to subtypes. The mapping of probe sets to ENTREZ gene identifiers, gene symbols, and KEGG [39] pathways was done using the *hgu133plus2.db* package (version 2.7.1). Presence/absence calls for probe sets were calculated from RMA expression by applying the *PANP* package [40]. We utilized the *genefilter* and the *multtest* packages to perform t-tests and Benjamini-Hochberg multiple testing correction, respectively. We utilized Fisher’s exact test to detect significant differences of clinical annotation between subtypes. To increase statistical power, we combined different datasets with the same annotation for this analysis. Samples contained in both datasets GSE14333 [26] and GSE17536 [41] were removed from GSE14333 for the combined analyses. Survival data was available for a total of 578 tumor samples from the sets GSE17536 [41], GSE17537 [41], GSE14333 [26], GSE33113 [42], and GSE37892. Staging was available for 488 of these samples and shows that roughly 75% are classified in intermediate stages (Additional file 1: Table S3). The survival time analysis was performed using the *survival* package. We censored survival data at follow-up time of 120 months because the number of samples with longer follow-up was small.

### Non-negative matrix factorization (NMF)

**Figure 2:**
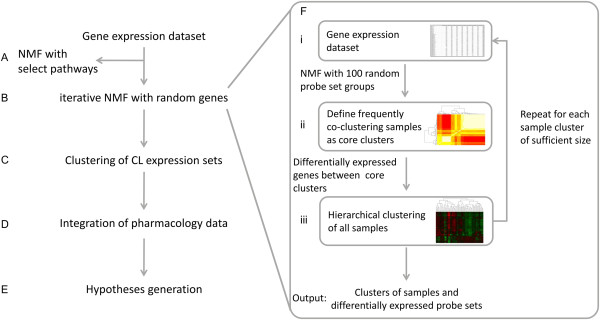
**Overview of the workflow followed in this work (left) and the proposed iterative Nonnegative Matrix Factorization (iNMF) clustering approach (right).** (**A**) First, we clustered a dataset consisting of 62 CRC samples using NMF based on four selected pathways. (**B**) Then, we applied iNMF for stratifying the samples with an unbiased selection of probe sets and (**C**) matched CRC cell lines (CL) to the resulting clusters. (**D**) By overlaying matching pharmacology data, (**E**) we investigated the potential for generating testable hypotheses regarding response of cell lines in different clusters.

We employed the *NMF* R-package [43] to perform non-negative matrix factorization (see Additional file 1) and the iterative NMF (iNMF, Figure 2). With each iteration of iNMF, sample clusters become more homogeneous in their expression. Therefore, it is possible to detect more subtle expression differences and achieve a more detailed subtyping. By applying NMF to many randomly selected probe set groups, iNMF achieves a stable sample clustering to detect so-called core clusters. The genes that serve as ‘signature’ genes for the subtypes are significantly differentially expressed between pairs of core clusters representing the subtypes. As input for NMF and iNMF, we used RMA normalized log_2_ expression values without mean-centering. We utilized 100 random probe set groups to carry out the iNMF analysis (see Additional file 1 for details). For each of those groups, we determined the optimal number of clusters using the cophenetic correlation coefficient (see Additional file 1 for details) and chose the most frequently selected number of clusters. Then, we calculated how often two samples co-clustered using the 100 random probe set groups. We determined core clusters of samples (Additional file 1: Table S4) based on a hierarchical clustering of this matrix but required samples to co-cluster at least 80 times. To detect differentially expressed genes, we compared expression of the 5000 genes with the highest variance between two single core clusters using the *t*-test and defined probe sets as differentially expressed if they had a Benjamini-Hochberg corrected FDR < 0.01. Using these probe sets, all samples in the input set were hierarchically clustered into the number of clusters determined by iNMF, thereby assigning the samples to the subtypes represented by these clusters. The hierarchical clustering agreed fully with the assignment of samples to core clusters. The resulting sample clusters were individually used as input set for the second iteration of the algorithm, following the same steps as outlined above to define the second level subtypes.

### INMF expression signatures

We detected 2154 probe sets (1351 genes) and 596 probe sets (408 genes) to be significantly up-regulated in Types 1 and 2, respectively (Additional file 2: Table S5 and Additional file 3: Table S6). Subsequently, Type 1 was split into three subtypes, 1.1, 1.2, and 1.3 that are defined by up-regulation of 439 probe sets (287 genes) for Subtype 1.1, 193 probe sets (141 genes) for Subtype 1.2, and 352 probe sets (219 genes) for Subtype 1.3 (Additional file 4: Table S7, Additional file 5: Table S8 and Additional file 6: Table S9). By further subdividing Type 2, we identified subtypes 2.1 and 2.2 with gene signatures consisting of 298 probe sets (200 genes) and 304 probe sets (202 genes), respectively (Additional file 7: Table S10 and Additional file 8: Table S11).

### EMT signature

We assembled an EMT expression signature by combi-ning two published EMT signatures [32,44], with genes from the SABiosciences EMT PCR array (SABiosciences, Frederick, MD). We annotated the genes as down- or up-regulated during EMT according to the source and removed genes with conflicting expression changes between different sets. In all cases, gene symbols were translated to probe set identifiers.

### Functional analysis

We performed a functional analysis of the subtype signatures using Signaling Pathway Enrichment using Experimental Datasets (SPEED) [45], and enrichment analyses on the Molecular Signatures Database (MSigDB) [46], and KEGG [39] and Pathway Interaction Database (PID) [47] using BioMyn [48]. Briefly, SPEED calculates enrichment of a gene list with signatures of downstream targets of selected pathways that were derived from pathway perturbation experiments. A significant overlap with a signature of a given pathway suggests that this pathway is activated. MSigDB contains gene sets divided into five collections: positional, curated, motif, computational, and GO; we calculated the overlap between the lists of genes that are differentially expressed between subtypes to the gene sets in all but the computational collection.

### Comparison to published CRC subtype gene expression signatures

We extracted the expression signatures published by Loboda *et al.*[32] and Oh *et al.*[33] and applied them to the datasets GSE2109 (provided by the Expression Project for Oncology of the International Genomics Consortium), GSE14333, GSE17536, and GSE17537. To this end, we calculated for each sample the difference between mean expression of the mesenchymal signature and the epithelial signature defined by Loboda and colleagues. Also, we subtracted for each sample the mean expression of genes up-regulated in type A from the mean expression of genes up-regulated in type B as defined by Oh and colleagues. Additionally, we determined expression of genes contained in the stromal signature published by Perez-Villamil *et al. *[34].

### Drug treatment

For measuring drug response in the AZCL panel, cell lines were maintained in the logarithmic phase of growth. The anti-proliferative activity of compounds was measured as EC50 values at 72 h after drug dosing using the MTS tetrazolium dye method (Promega), proliferation assays were seeded at appropriate density to ensure logarithmic growth during the 72 h dosing period. For each compound, the mean -log_10_ (EC50) was computed across all cell lines and subtracted from -log_10_ (EC50) value for each cell line. The resulting value is positive if a cell line is more sensitive to treatment with this compound than the average over all lines and negative if it is more resistant. For the Sanger cell line panel, we downloaded IC50 values provided by the Cancer Genome Project group at the Wellcome Trust Sanger Institute from http://www.cancerrxgene.org/translation/Drug on June 6, 2012 [16]. As for the AZCL set, we calculated the average -log_e_(IC50) for each compound across all cell lines and subtracted this value from -log_e_ (IC50) for each cell line. The resulting value is positive if a cell line is more sensitive than average to treatment with a specific compound.

## Results

### Five CRC subtypes are revealed by iterative clustering

For a pathway-based stratification of CRC tumor samples, we selected four pathways known to play a role in progression of CRC: MAPK signaling (KEGG: hsa04010), mTOR signaling (KEGG: hsa04150), ErbB signaling (KEGG: hsa04012), and colorectal cancer disease pathway (CRCdp, KEGG: hsa05210). Applying NMF independently to the gene sets annotated to these four pathways resulted in two sample clusters that were overlapping significantly for the ErbB, MAPK, and CRCdp pathways, indicating dominant gene expression differences involving these pathways.

**Figure 3:**
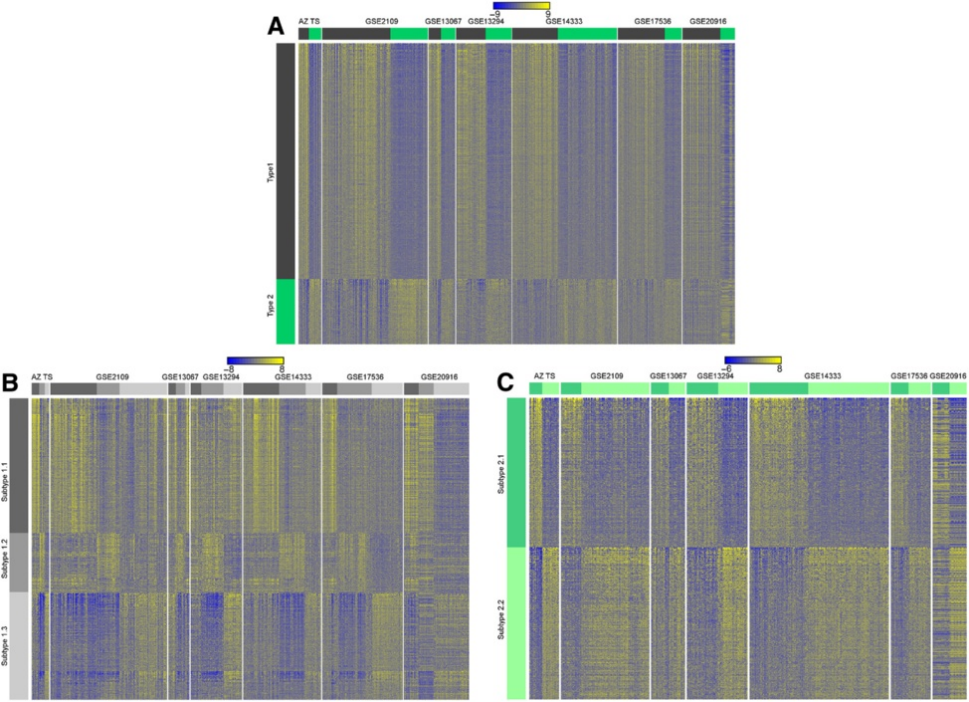
**Expression patterns of CRC subtypes defined by iNMF in different datasets: (A) Types 1 (black) and 2 (green) from iNMF iteration 1; (B) Subtypes 1.1, 1.2, and 1.3; and (C) Subtypes 2.1 and 2.2.** Samples are shown in columns and probe sets contained in the subtype signatures are shown in rows.

Next, we applied iterative NMF (iNMF) to 100 randomly selected groups of probe sets, roughly equal in size to these pathways. See Figure 2 for a schematic representation of the iNMF procedure. The first iteration of iNMF resulted in two sample clusters (Types 1 and 2). In the second iteration, Type 1 was split into three subtypes, denoted as Subtypes 1.1, 1.2, and 1.3, whilst application of iNMF to Type 2 led to the identification of Subtypes 2.1 and 2.2 (Table 2 and Figure 3). The gene signatures are listed in Additional file 2: Table S5, Additional file 3: Table S6, Additional file 4: Table S7, Additional file 5: Table S8, Additional file 6: Table S9, Additional file 7: Table S10, Additional file 8: Table S11 for the respective subtypes. When we applied iNMF to 10.000 additional randomly selected probe set groups, the core clusters did not change significantly showing that the discovered subtypes do not depend on the randomly selected probe set groups.

**Table 2:** Comparison of clinical and molecular characteristics of identified CRC subtypes

**iNMF step 1**					
**Molecular feature**	**Type 1**			**Type 2**	
No. of tumor samples (AZTS)	28			34	
No. of tumor samples (all datasets)	944			762	
EMT expression signature	mesenchymal			epithelial	
Survival prognosis	poor			good	
Microsatellite status	similar number of MSI and MSS CRC			enriched with MSS CRC	
**iNMF step 2**					
**Molecular feature**	**Subtype 1.1**	**Subtype 1.2**	**Subtype 1.3**	**Subtype 2.1**	**Subtype 2.2**
No. of tumor samples (AZTS)	12	9	7	14	20
No. of tumor samples (all datasets)	313	303	328	313	449
Average percent tumor foci area	79.2	81.4	77.9	82.1	79.7
Average percent tumor cells in foci	76.3	74.5	80.0	73,8	73.7
Average percent stromal cells in foci	18.3	18.3	12.2	21.2	21.2
Average percent inflammatory cells in foci	3.8	7.2	6.4	3.5	4.7
EMT expression signature	strongly mesenchymal	mesenchymal	mesenchymal	epithelial	epithelial
Tumor location colon/left/rectum/right	0/26/16/15	1/12/1/29	0/12/2/11	1/22/9/37	1/50/11/33
Microsatellite status MSI/MSS	14/15	42/9	2/35	27/31	4/50
Tumor stage	enriched in late stage CRC				
Gender enrichment Female/Male/Unknown	87/99/0	89/57/0	90/124/1	58/34/0	108/113/0
Up-regulated genes	Ca-signaling and SRF-targeted	immune system-related	transporters	stress response and immune system-related	cell cycle and amino acid synthesis; genes on 13q13, 13q14, 13q32-34 and 20q11and 20q13

Differences in these characteristics were assessed using all datasets with available annotation.

### The CRC subtypes are present in independent datasets

In order to further establish the robustness of the iNMF method and the resulting stratification, we performed two rounds of validation. First, we hierarchically clustered several independent, publically available CRC expression datasets totaling 1643 samples (Additional file 1: Table S1) with the probe sets that were found to be differentially expressed between subtypes in our dataset. Additional file 9: Table S12 summarizes the results. Figure 3 depicts an overview of the expression signatures across the biggest datasets. It is clear that we were able to identify all subtypes in external datasets. More importantly, the expression of the signature genes is consistent across the different datasets.

Second, we applied iNMF to a large independent set GSE14333 (n = 290) and investigated the overlap of the resulting stratification with the one obtained from the first validation. We utilized the same groups of randomly selected probe sets and performed two iterations of iNMF. The first iteration identified very similar CRC Types 1 and 2 as before (Fisher exact p-value < 2.2*10^-16^), and 68% and 71% (p-values < 9.9*10^-16^) of the genes that were previously found to be up-regulated in CRC Types 1 and 2, respectively. The subtypes identified in the second iteration were also significantly similar (simulated Fisher exact p-value = 5*10^-4^). The overlaps of gene signatures were significant for subtypes of Type 1 and for Subtype 2.1 (p-values < 8.6*10^-8^) but not for 2.2.

### CRC subtypes exhibit significantly different molecular and clinical characteristics

To gain insight into the correlation of the iNMF stratification with available clinical annotation, we made use of the annotation available for some datasets. Here, it has to be noted that clinical annotation varies substantially between different datasets. Table 2 summarizes the differences in clinical characteristics.

Survival analysis revealed that Type 1 had significantly worse disease free survival (p-value = 9*10^-3^) than Type 2 (Additional file 1: Figure S1). Using survival and chemotherapy annotation of samples in GSE14333, an univariate Cox regression model indicated that Stage C tumors assigned to Type 1 had a significantly improved disease free survival if treated with chemotherapy (p-value = 0.04) while Stage C tumors in Type 2 did not show such a benefit. Furthermore, there was also a significant difference in the distribution of MSI samples (p-values < 8.68*10^-4^) between Subtypes 1.1, 1.2 and 1.3. The three subtypes of Type 1 also showed significant differences in terms of distribution of tumor location (p-value = 3.42*10^-3^). Of note, there was also a significant difference in distribution of male and female samples (p-value = 1.12*10^-3^) with Subtype 1.2 being the only subtype comprising more tumors of female than male patients.

### Major CRC types exhibit a mesenchymal and an epithelial, cell cycle-activated profile

To further characterize the CRC subtypes at a functional level, we subjected the lists of subtype signature genes to a functional analysis using Signaling Pathway Enrichment using Experimental Datasets (SPEED) [45], the Molecular Signatures Database (MSigDB) [46], and KEGG [39] and Pathway Interaction Database (PID) [47] using BioMyn [48]. A detailed description of the results can be found in Additional file 1.

**Figure 4:**
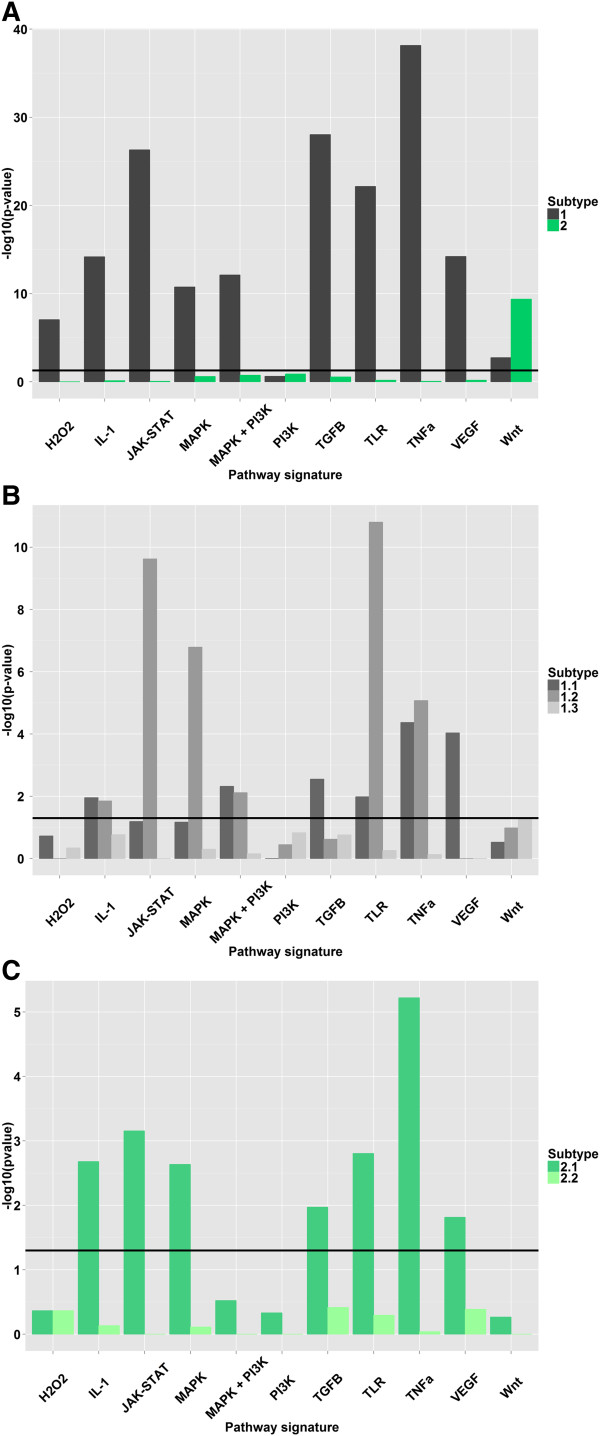
**Overview of SPEED analysis of pathway activation in (A) Types 1 and 2, (B) Subtypes 1.1, 1.2, and 1.3, and (C) Subtypes 2.1 and 2.2.** The y-axes denote negative logarithm to base 10 of the activation p-value. The horizontal lines indicate the significance threshold of p-value = 0.05.

For Type 1, we found a large number of pathways to be activated, which are related to inflammation, angiogenesis, extracellular matrix, proliferation, and differentiation (Figure 4A). In contrast, Type 2 can be characterized by activation of the Wnt pathway, up-regulation of cell cycle-related genes, including aurora kinase A. Since a number of pathways that have been linked to EMT are significantly more activated in Type 1, we performed a hierarchical clustering of the AZTS dataset using an EMT-related gene signature. This revealed a high concordance between stratification into Type 1 and 2 and mesenchymal and epithelial expression profiles (Additional file 1: Figure S2), respectively (Chi square p-value = 4.7*10^-10^). These results confirm previous evidence [32] that EMT is correlated with dominant expression changes in CRC.

### Subtypes show selective pathway activation

We also identified several pathways to be activated in the subtypes. Subtype 1.1 is characterized by pathways involved in angiogenesis, inflammation, and proliferation (Figure 4B). Intriguingly, we also found a significant up-regulation of the calcium signaling KEGG pathway (p-value = 0.01) in 1.1. Subtype 1.2 shares activation of many pathways with Subtype 1.1 but strong activation of JAK-STAT is unique to 1.2 (Figure 4B). In Subtype 1.3, we identified genes annotated with several Gene Ontology (GO) [49] terms related to transport across membranes (p-values < 0.05) to be up-regulated (Table 2).

In Subtype 2.1, we identified several activated pathways related to inflammation, angiogenesis, and proliferation(Figure 4C). Intriguingly, we identified a number of genes to be up-regulated in Subtype 2.2 from two cytogenetic bands on the q-arm of Chromosome 20 (20q11 and 20q13, p-values < 5.68*10^-5^), and several bands on Chromosome 13q (13q13-14, 13q32-34, p-values < 3.96*10^-2^).

### Comparison with published subtype signatures

**Figure 5:**
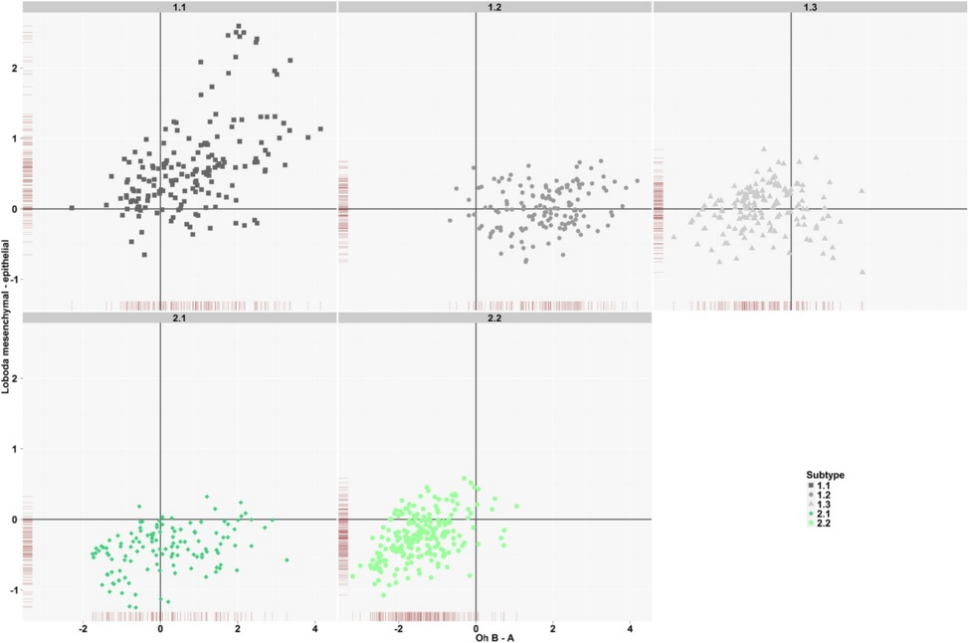
**Comparison between iNMF subtypes and subtypes identified by Loboda *et al. *and Oh *et al.*** Shown are samples contained in GSE2109, GSE14333, GSE17536, and GSE17537. The x- and y-axes depict the difference between average expression of signatures published by Oh *et al.* and Loboda *et al.* Lines along the axes represent the density of samples of the respective iNMF subtypes.

Recently, Loboda *et al.* showed that EMT represents a dominant gene expression signal in human CRC [32]. The mesenchymal and epithelial subtypes identified by Loboda and colleagues largely agree with the dominant iNMF Type 1 and 2. Oh *et al.* identified two subtypes that exhibit differences in survival and response to chemotherapy [33]. As shown in Figure 5, the two published signatures clearly detect different tumor samples and features of CRC. The iNMF subtyping reveals the extremes of these groups, high expression of both signatures, is associated with Subtype 1.1, whilst low expression of both signatures correlates with Subtype 2.2. In addition, the iNMF subtying combines the features of the two signatures to further discriminate CRC subtypes, e.g. Subtype 2.1 is epithelial with either low or high expression of the Oh type B signature. Recently, Perez-Villamil *et al.* identified four subtypes in CRC including a stromal, poor survival subtype. As shown in Additional file 1: Figure S3, genes in this stromal signature are mainly expressed in Type 1 samples indicating that the stromal signature cannot distinguish between the detailed subtypes identified by iNMF.

### CRC cell line panels represent all five subtypes

**Figure 6:**
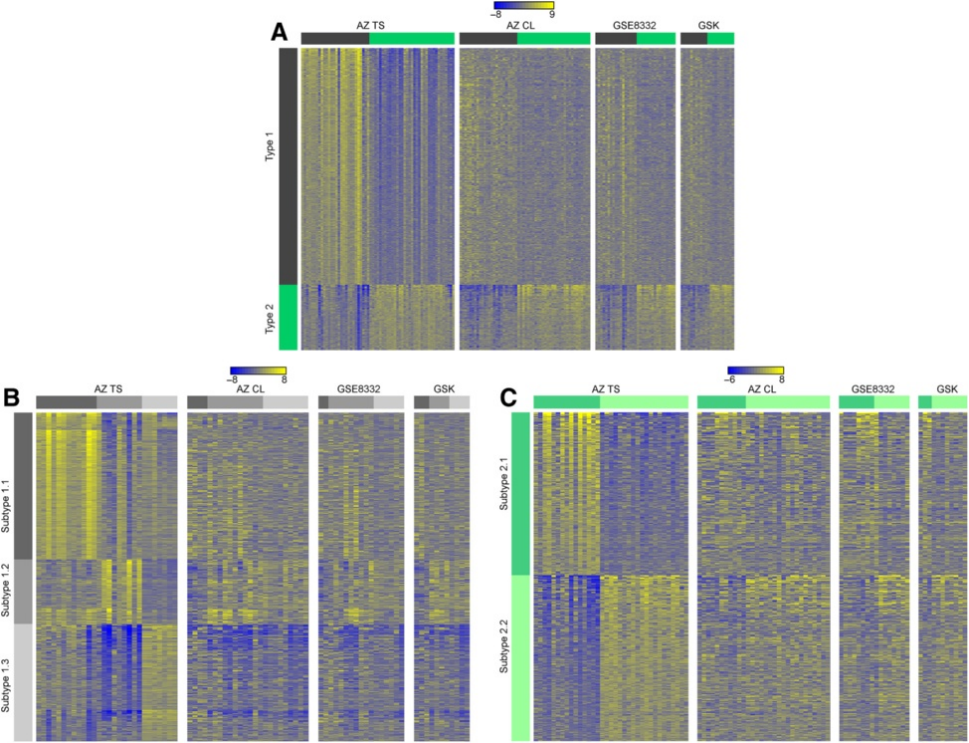
**Expression patterns of CRC subtypes defined by iNMF in the AZTS set and different cell line panels.** (**A**) Cell lines that are matched to Types 1 (black) and 2 (green) show a very similar expression pattern to the tumor samples. Expression in cell lines assigned to (**B**) the subtypes of Type 1 and (**C**) subtypes of Type 2 shows less similarity with expression in tumor samples.

In order to assess how well the identified CRC subtypes are represented in available cell line panels, we investigated four different datasets. First, we applied the subtype signatures obtained by iNMF using hierarchical clustering to a diverse, combined panel containing 67 CRC cell lines (AZCL, GSK, GSE8332). In general, the expression of signature genes in cell lines was less pronounced, but nevertheless, all subtypes were identified (Figure 6 and Additional file 1: Table S13). Furthermore, the alignment of cell lines to subtypes was consistent across datasets, SW480 being the only cell line showing inconsistent alignment. In general, we observed that the expression of genes pointing at activation of specific pathways in the SPEED analyses is less consistent in cell lines than in tumor samples. The genes indicating activation of Jak-Stat signaling in tumors in Subtypes 1.2 and 2.1, for instance, are not consistently expressed in the cell lines assigned to these subtypes. However, the average expression of all genes in the SPEED Jak-Stat signature is significantly higher in 1.2 cell lines than in 1.1 and 1.3 cell lines (Wilcoxon rank sum p-values < 0.002) indicating that the pathway activation may be conserved between primary tumors and cell lines in the same subtype. Furthermore, expression of EMT markers in cell lines shows a similar pattern as in tumor samples (Additional file 1: Figure S4).

Last, we applied the subtype signatures to the Sanger dataset, comprising 34 cell lines that were profiled on a different expression platform (Additional file 1: Table S14). The stratification of cell lines overlapping between the Sanger set and the other cell line panels was highly similar (p-value = 8.6*10^-6^).

### Differential response of CRC subtypes to targeted inhibitors

**Figure 7:**
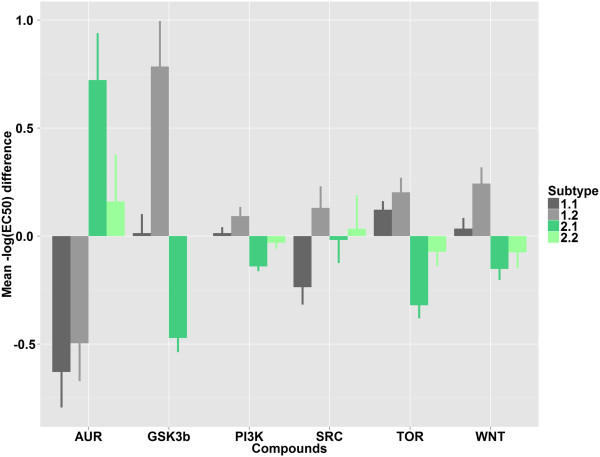
**Pharmacological response of cell lines in the AZCL panel to targeted inhibition.** The y-axis denotes difference between average –log_10_ (EC50) of cell lines assigned to one subtype and average –log_10_ (EC50) of all measurements for compounds targeting the indicated protein. Positive or negative values indicate that cell lines in a cluster are more sensitive or resistant, respectively, than the overall average. Standard error of subtype means are represented as lines.

**Figure 8:**
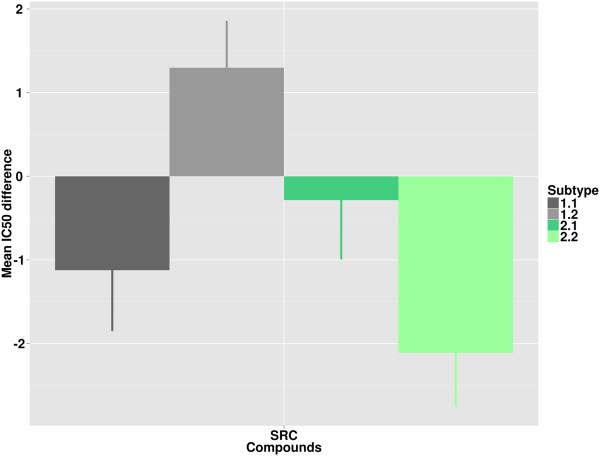
**Pharmacological response of cell lines in the Sanger panel to targeted inhibition.** The y-axis denotes difference between average –log_10_ (IC50) of cell lines assigned to one subtype and average –log_10_ (IC50) of all measurements for compounds targeting the indicated protein. Positive or negative values indicate that cell lines in a cluster are more sensitive or resistant, respectively, than the overall average. Standard error of subtype means are represented as lines.

To assess the potential clinical utility of the subtypes, we determined whether cell lines assigned to different subtypes respond differently to targeted inhibitors. To this end we determined the association between pharmacological response data available for part of the AZCL set (Figure 7, Additional file 10: Table S15) and the Sanger set with the subtyping (Figure 8, Additional file 1: Table S16). These two independent datasets overlap partly in terms of cell lines. In both cases, we pooled compounds with the same target for this analysis.

Aurora kinase A was one of the genes significantly up-regulated in Type 2. In accordance with this, cell lines assigned to Type 2 are significantly more sensitive to treatment with targeted aurora kinase inhibitors than cell lines of Type 1 (p-value = 1.9*10^-3^). Furthermore, cell lines assigned to Subtype 1.2 show a specifically high sensitivity to treatment with inhibitors of glycogen synthase kinase, the proto-oncogene tyrosine-protein kinase Src, and Wnt-signaling.

The analysis of the Sanger pharmacology data provides further validation for the high sensitivity of Subtype 1.2 cell lines to targeted inhibition of Src. As for the AZCL dataset, cell lines assigned to 1.2 exhibit the highest average sensitivity to these compounds.

## Discussion

In this work, we introduced iterative nonnegative matrix factorization based on randomly selected probe sets and applied it for stratifying CRC samples in a two-step process into two main types and subsequently into five subtypes. In contrast to previous studies, this iterative process enables us to detect a hierarchical relationship between subtypes based on expression differences of varying strength. Being based on randomly selected probe sets, iNMF has the advantage that it is unbiased with respect to knowledge about genes and pathways. The subtype signatures consisting of differentially expressed probe sets can be easily applied for hierarchically clustering independent CRC datasets in a two step process, thereby assigning the samples to their respective subtypes.

The presented CRC stratification was validated by clustering independent CRC expression datasets using the identified signatures and by applying the iNMF algorithm to an independent dataset, which resulted in highly similar subtypings. These results prove that our method and stratification are robust and transferable to other datasets, and that the lists of differentially expressed probe sets are applicable for the stratification of independent expression datasets and robust against confounding factors typically present in independent datasets.

The functional analyses of differentially expressed probe sets provided insight into differences in the activation of key signaling pathways in distinct types and subtypes and interesting start points for further investigations. The first iteration revealed a mesenchymal (Type 1) and a highly proliferative, epithelial (Type 2) type. This difference between epithelial and mesenchymal types is not correlated to the amount of infiltration by stromal cells as tumor sampels in all subtypes show similar percent stromal cells (Table 2). Further stratifying the mesenchymal type identified a subtype with signs of activation of MAPK, TGFβ, and calcium signaling (Subtype 1.1), a subtype with activation of immune system-related pathways (Subtype 1.2), and one with high expression of transporter genes (Subtype 1.3). The subdivision of the epithelial type revealed a subtype showing activation of immune system-related pathways (Subtype 2.1), and a subtype with high expression of genes on chromosomes 13q and 20q (Subtype 2.2).

Many of the pathways identified here as activated in specific subtypes were also shown to be targeted by recurrent alterations in a recent analysis by The Cancer Genome Atlas Network [50]. In this analysis, most samples were found to harbor alterations leading to an activation of WNT signaling which is in agreement with the finding that WNT is the only pathway analyzed that seems to be activated in both Types 1 and 2. Furthermore, receptor tyrosine kinase-RAS signaling was affected in a substantial number of tumors, and we identified classical MAPK signaling to be activated in Type 1 and specifically in Subtype 1.1. Recently, Seshagiri and colleagues analyzed next-generation sequencing data obtained from 70 primary human colon tumors [51] and found frequent mutations in 356 candidate CRC genes previously identified in screens in mouse models of CRC [52,53]. More than 8% of these genes are also contained in the signatures associated with the iNMF subtypes presented here. Clusterin, for example, is highly expressed in Type 1 and known to regulate NF-κB activity [54] and inhibit apoptosis [55]. Type 2 tumors, on the other hand, show high expression of dachshund homolog 1 which inhibits TGFβ signaling through binding to SMAD4 [56] and possibly contributes to the difference in TGFβ signaling between Type 1 and 2. This provides further evidence that the iNMF signatures and the differences in pathway activation between subtypes represent CRC intrinsic features and contribute to their better understanding.

Subtype 1.2 is highly enriched for tumors showing MSI, which have been shown to have substantial amounts of tumor-infiltrating lymphocytes [57]. Although the average percent of infiltrating inflammatory cells is comparable across subtypes (Table 2), Subtype 1.2 indeed shows the highest average and this might have influenced the gene expression signatures. Unexpectedly, Subtype 1.2 is the only subtype that comprises more female than male tumors. Previously, it has been reported that there are differences regarding the location distribution of colorectal tumors between the genders, e.g. that in women right-sided CRC is more common [58] and that pathological and molecular features of the tumors vary between locations [59]. These variations might cause changes in gene expression which are detected by iNMF.

Aligning cell lines with tumor samples to enhance their utility as pre-clinical predictive models has proved challenging for many tumor types. We observed that the four cell line panels investigated here generally provided a good coverage of the space of primary tumor samples, in contrast to a study by Auman and McLeod [60]. Although the expression of the signature genes is less consistent in cell lines, replicates from different panels were stratified in a highly consistent fashion. Furthermore, specific biological characteristics agreed between tumor samples and cell lines assigned to the same subtype. The observation that expression patterns for the pathways investigated are not well conserved between cell lines and tumor samples might indicate that canonical pathways do not fully reflect mechanistic complexity. Furthermore, the non-natural culture conditions of cell lines might have an effect on gene expression which might change the activation of pathways or the respective expression signal that can be detected. However, the successful alignment of CRC cell lines to the newly identified disease subtypes using the techniques described here reveals that the gene expression profiles which define subtypes remain significantly intact despite extended growth in vitro.

Analysis of two cell line datasets with treatment response data indicated that subtypes respond differently to targeted compounds. Type 2 cell lines are more sensitive to treatment with aurora kinase inhibitors. This is in agreement with the high expression of aurora kinase A in Type 2 tumor samples and suggests that genes included in the signatures might be good candidates for targeted treatment of specific CRC subpopulations. Additionally, pharmacological data for two independent cell line panels suggests that Subtype 1.2 cell lines are most sensitive to inhibition of Src. These are interesting hypotheses for the treatment of the different CRC subtypes that warrant further investigation.

The comparison to published signatures showed that the five iNMF subtypes are neither detected by any of the existing signatures alone nor by their combination. For example, most tumors in Subtype 1.2 and many tumors in 2.1 have a high Oh B signature but differ in EMT status. Interestingly, Subtype 1.2 shows a significantly higher sensitivity than Subtype 2.1 to inhibition of proteins on the PI3K pathway, GSK3β, PI3K, and TOR. This suggests that the subtyping presented here allows a more fine grained subdivision of CRC samples which is likely to have greater utility at linking molecular features to pharmacology.

## Conclusions

In summary, we have used tumor gene expression profiles to identify new CRC subtypes and have defined their main pathway differences. Using a large number of independent datasets, we showed that the stratification is stable across different datasets, regardless of which dataset is employed to derive the gene sets with which to perform the stratification. iNMF is robust with respect to the starting dataset and can be applied to identify inherent disease subtypes. Furthermore, we have presented evidence that CRC cell line panels represent the different disease subtypes, and that the integration of pharmacology data offers new opportunities for develo-ping improved CRC therapies targeted at the new CRC molecular subtypes and generating clinically tractable hypotheses for response prediction.

## Competing interest

Andreas Schlicker has received funding from Astra Zeneca Garry Beran is an employee of Astrazeneca Christine M Chresta is an employee of Astrazeneca Gael McWalter is an employee of Astrazeneca Alison Pritchard is an employee of Astrazeneca Susie Weston is an employee of Astrazeneca Sarah Runswick is an employee of Astrazeneca Sara Davenport is an employee of Astrazeneca Kerry Heathcote is an employee of Astrazeneca Denis Alferez Castro is an employee of Astrazeneca George Orphanides is an employee and shareholder of Astrazeneca Tim French is an employee and shareholder of Astrazeneca Lodewyk FA Wessels has received funding from Anstra Zeneca.

## Authors’ contributions

AS, GB, CMC, GO, TF, and LFAW participated in the design and performance of the study and in the analysis and interpretation of the data. GM, AP, SW, SR, SD, KH, and DAC participated in generation of the data. The manuscript was drafted by AS, GB, CMC, GO, TF and LFAW and reviewed by all authors. All authors read and approved the final manuscript.

## Authors’ information

Tim French and Lodewyk FA Wessels shared last authorship.

## Pre-publication history

The pre-publication history for this paper can be accessed here:

http://www.biomedcentral.com/1755-8794/5/66/prepub

## Supplementary Material

Additional file 1 **Supplementary analysis and results.** Tables S1-S4, S13-S14, S16, and Additional file 1: Figure S1, Additional file 1: Figure S2, Additional file 1: Figure S3 and Additional file 1: Figure S4. Click here for file

Additional file 2 **Table S5.** Probe sets contained in the Type 1 gene signature. Click here for file

Additional file 3 **Table S6.** Probe sets contained in the Type 2 gene signature. Click here for file

Additional file 4 **Table S7.** Probe sets contained in the Subtype 1.1 gene signature. Click here for file

Additional file 5 **Table S8**. Probe sets contained in the Subtype 1.2 gene signature. Click here for file

Additional file 6 **Table S9.** Probe sets contained in the Subtype 1.3 gene signature. Click here for file

Additional file 7 **Table S10.** Probe sets contained in the Subtype 2.1 gene signature. Click here for file

Additional file 8 **Table S11.** Probe sets contained in the Subtype 2.2 gene signature Click here for file

Additional file 9 **Table S12.** Results of stratifying different colorectal cancer datasets. Click here for file

Additional file 10 **Table S15.** Pharmacological response data for cell line panel AZCL. Click here for file
