# Supplementary material for: Subtypes of primary colorectal tumors correlate with response to targeted treatment in colorectal cell lines
Source: BMC Med Genomics. 2012 Dec 31;5:66. doi: 10.1186/1755-8794-5-66 (PMC3543849; doi:10.1186/1755-8794-5-66)
Supplement: Additional file 1 — Supplementary analysis and results. Tables S1-S4, S13-S14, S16, and Additional file 1: Figure S1, Additional file 1: Figure S2, Additional file 1: Figure S3 and Additional file 1: Figure S4. [file 1755-8794-5-66-S1.doc]

**Supplementary Data**

**Subtypes of primary colorectal tumors correlate with response to targeted treatment in colorectal cell lines**

Andreas Schlicker^1^, Garry Beran^3^, Christine M Chresta^3^, Gael McWalter^4^, Alison Pritchard^3^, Susie Weston^4^, Sarah Runswick^4^, Sara Davenport^3^, Kerry Heathcote^3^, Denis Alferez Castro^3^, George Orphanides^3^, Tim French^4,*^, Lodewyk FA Wessels^1,2,*^

^1^ Division of Molecular Carcinogenesis, The Netherlands Cancer Institute, Amsterdam, The Netherlands ^2^ Faculty of EEMCS, Delft University of Technology, Delft, The Netherlands ^3^ Oncology iMed, AstraZeneca R&D, Alderley Park, Macclesfield, United Kingdom ^4^ Personalised Healthcare and Biomarkers Group, AstraZeneca R&D, Alderley Park, Macclesfield, United Kingdom ^*^shared last authorship

**Nonnegative matrix factorization**

Nonnegative matrix factorization (NMF) is an unsupervised clustering method that has previously been applied for stratifying samples and genes based on gene expression [1]. NMF factorized the expression matrix *V* into two nonnegative matrices *W* and *H* such that *V~ WH*. Rows of *W* represent metagenes that are defined as nonnegative linear combinations of genes in the input expression matrix. Similarly, columns in *H* describe expression patterns of the metagenes in the corresponding sample. A parameter determines the number of clusters.

For one NMF run, matrices *W* and *H* are initialized randomly. Therefore, the algorithm is repeated several times to achieve a stable clustering. The consensus matrix summarizes how often two samples have been clustered together in the different repeats. This consensus matrix can then be utilized for estimating the optimal number of clusters (rank k) based on the cophenetic correlation coefficient. This coefficient is defined as the correlation between the sample distances induced by the cophenetic distances obtained by hierarchical clustering of the samples and the consensus matrix.

To estimate the number of clusters, we repeated NMF 30 times for different possible values. As suggested by Brunet *et al*., the final rank was estimated as the smallest number for which the cophenetic correlation coefficient started to decrease [2]. Finally, NMF was repeated 100 times with defined rank k to obtain the clustering of samples and probe sets.

**Pathway-restricted nonnegative matrix factorization**

Pathway-restricted NMF was performed for four KEGG pathways: MAPK, ErbB, mTOR, and the colorectal cancer disease pathway (CRCdp). All probe sets annotated to these pathways were selected; probe sets were discarded if they were called absent in all 62 tumor samples. We performed NMF [1] on the resulting sets of 438, 99, 165, and 162 probe sets representing MAPK, mTOR, ErbB, and CRCdp, respectively, using the NMF R-package [3].

Figure 1 depicts an overview of the iterative NMF (iNMF) approach. First, we created 100 random groups of probe sets with size similar to the MAPK pathway set. Probe sets that were called absent in all 62 tumor samples or were mapped to one of the pathways analyzed in the pathway-restricted NMF were excluded. Second, NMF analysis was carried out using the 100 random probe set groups. Third, core clusters consisting of samples that frequently co-clustered in the NMF results for the 100 random probe set groups were determined. Probe sets were defined as being differentially expressed between two clusters if they had a t-test p-value < 0.05 and Benjamini-Hochberg corrected FDR < 0.01. Fourth, all samples in the input set were subjected to hierarchical clustering based on the list of differentially expressed probe sets from the previous step. The resulting sample clusters were individually used as input set for the next iteration of the algorithm.

**Pathway-restricted NMF**

**MAPK**

NMF of the MAPK pathway revealed that samples in cluster 1 show up-regulation of a number of genes whose products are involved in calcium-signaling or are calcium-regulated: membrane associated, cytosolic, and calcium-dependent phospholipase A2 (PLA2G2A, PLA2G4A, and PLA2G5), the voltage-dependent calcium channel beta 2 subunit (CACNB2), and the RAS guanyl-releasing protein 1 and 3 genes (RASGRP1 and RASGRP3, respectively). Single-span transmembrane glycoproteins such as E-cadherins are known to interact in a calcium-dependent manner to form adherens junctions across cells [4]. Furthermore, filamin A and C (FLNA and FLNC, respectively) are up-regulated, which provide a link between the actin cytoskeleton and a number of signaling proteins. Importantly, actin cytoskeleton remodeling is one of the hallmarks of EMT [4]. Transforming growth factor beta 1 and 3 (TGFB1 and TGFB3, respectively) are found to be over-expressed in this sample cluster and are known to be specific for the induction of EMT [5]. Additionally, transforming growth factor beta receptor 1 (TGFBR1) is found to be up-regulated.

In cluster 2, subunit alpha-1D of the voltage-dependent L-type calcium channel (CACNA1D) is up-regulated as well as protein kinase C alpha (PRKCA). PRKCA contains a C2 domain that binds three calcium ions and is known to initiate cell polarization and motility [6]. Interestingly, mitogen-activated protein kinase 13 (MAPK13, also known as p38δ) is up-regulated along with several genes that are involved in the JNK/p38 branch of MAPK signaling, including the serine/threonine-protein kinases PAK1 and PAK2, the p38 phosphorilation target MAP kinase-activated protein kinase 3 (MAPKAPK3), dual specificity mitogen-activated protein kinase kinase 6 (MAP2K6), dual specificity protein phosphatase 16 (DUSP16), mitogen-activated protein kinase kinase kinase MLT (MLTK, also known as ZAK), and MECOM. P38δ has been shown to engage in a complex with ERK1/2 and to directly inhibit ERK1/2 function [7]. MECOM (also known as EVI1) is a known oncoprotein that inhibits Smad3 and thereby represses TGF-beta signaling [8]. This is in contrast to genes over-expressed in cluster 1 in which several TGF-beta signaling proteins are over-expressed. Furthermore, MECOM has been shown to counteract stress-induced cell death by inhibition of c-Jun N-terminal kinase (JNK) [9], and TGF-beta induced apoptosis via a PI3K/AKT signaling cascade [10].

**ErbB**

Samples in cluster 1 show over-expression of PI3-kinase catalytic subunit delta (PI3KCD) and the PI3-kinase regulatory subunit 5 (PI3KR5) as well as RAC-alpha serine/threonine-protein kinase 3 (AKT3). Moreover, these samples show up-regulation of protein kinase C beta (PRKCB) and phospholipase C gamma 2 (PLCG2), which are both connected to calcium signaling. Interestingly, cluster 2 of the ErbB pathway is characterized by high expression of amphiregulin (AREG) and epiregulin (EREG), which are both members of the epidermal growth factor (EGF) family and bind to the epidermal growth factor receptor (EGFR/ErbB1). Patients with high AREG and EREG expression have been previously found to better respond to Cetuximab treatment [11] but also to have a worse prognosis [12].

**CRCdp**

Samples in cluster 1 of the CRCdp show up-regulation of a number of different Frizzled receptors (FZD1, FZD2, FZD4, and FZD7), which in mouse are known to bind members of the PSD-95 protein family [13]. Furthermore, FZD2 forms ternary complexes with PSD-95 and adenomatous polyposis coli protein (APC), which is frequently mutated in CRC [14]. Comparable to the results for the MAPK pathway, several members of (TGF-ß) signaling were found to be over-expressed in this sample cluster (TGFB1, TGFB2, TGFB3, and TGFBR1). Alpha- and beta-type platelet-derived growth factor receptors (PDGFRA and PDGFRB) are also up-regulated in samples in cluster 1. Intriguingly, it has been shown that PDGF increases EMT in colon cancer cells by translocation of ß-catenin in a Wnt-independent manner [15]. Moreover, the transcription factor lymphoid enhancer-binding factor 1 (LEF1) is found to be up-regulated, which is frequently up-regulated in B-cell chronic lymphocytic leukemia (CLL) [16]. LEF1 has recently been shown to be a promising target to disrupt Wnt/ß-catenin signaling in the context of CLL treatment [16]. Additionally, LEF1, a target of TGF-ß signaling, possesses binding sites for Smad proteins and ß-catenin and is strongly implicated in EMT [17]. Mutations in the APC gene, which frequently occur in CRC, and the resulting accumulation of ß-catenin can also lead to the formation of a complex consisting of LEF1 and ß-catenin in a Wnt-independent fashion [17].

On the contrary, some samples in cluster 2 over-expressed FZD5 and FZD10, survivin (BIRC5), AXIN2, and the DNA mismatch repair proteins MSH2 and MSH6. Survivin is a member of the inhibitor of apoptosis family and a direct target of Wnt signaling that is up-regulated by ß-catenin [18]. Survivin is thought to confer a stem-cell-like phenotype to CRC cells when over-expressed through a ß-catenin-linked mechanism [19]. The DNA mismatch repair complex MutS alpha is a heterodimer consisting of Msh2 and Msh6, which is involved in the repair of single base pair mismatches and dinucleotide insertion-deletion loops [20, 21]. These two genes are known to be frequently mutated in microsatellite instable (MSI) CRC [14, 22]. The AXIN2 gene found to be over-expressed in samples in this cluster is another component of the canonical Wnt-signaling pathway which has been associated with defects in DNA mismatch repair in CRC [14, 23].

### Major CRC types exhibit a mesenchymal and an epithelial, cell cycle-activated profile

To further characterize the CRC subtypes at a functional level, we subjected the lists of subtype signature genes to a functional analysis using Signaling Pathway Enrichment using Experimental Datasets (SPEED) [24], the Molecular Signatures Database (MSigDB) [25], and KEGG [26] and Pathway Interaction Database (PID) [27] using BioMyn [28].

For Type 1, SPEED indicated a large number of pathways to be activated, including JAK-STAT, TNF-α, TGF-ß, and TLR (Figure 3A). Enrichment analysis on MSigDB revealed that many genes induced in Type 1 were annotated as being regulated by the serum response factor (SRF), a downstream effector of classical MAPK signaling (p-values < 2.3*10-5). There was also a significant fraction of genes that were annotated with GO terms related to the extracellular matrix (p-values < 4.94*10-5) and immune response (p-value = 4.25*10-5). The KEGG pathway analysis largely confirmed these findings. More specifically, genes upregulated in Type 1 had a highly significant overlap with the KEGG pathways ‘cell adhesion molecules’ (p-value = 1.39*10-10), ‘ECM-receptor interaction’ (p-value = 1.61*10-10), and ‘focal adhesion’ (p-value = 2.02*10-9).

In contrast to Type 1, the SPEED analysis revealed significant activation of only the Wnt pathway in Type 2 (Figure 3A). Genes up-regulated in Type 2, also showed a significant overlap with cell cycle-related GO terms (p-values < 1.8*10-3), including Aurora kinase A, and significant overlap with genes that are regulated by the transcription factors E2F1 and TFDP1 (p-values < 0.05). These genes also overlapped significantly with the “cell cycle” KEGG pathway (p-value = 1.71*10-6), and the PID pathways “Aurora B signaling” (p-value = 3.1*10-3) and “E2F transcription factor network” (p-value = 3.5*10-3).

A number of pathways that have been linked to EMT, including TNF-α, TGF-ß, and MAPK [19, 29, 30], are significantly more activated in Type 1. Hierarchical clustering of the AZTS dataset using an EMT-related gene signature revealed a high concordance between stratification into Type 1 and 2 and mesenchymal and epithelial expression profiles (Figure S2), respectively (Chi square p-value=4.7*10-10). This confirms previous evidence [31] that EMT is correlated with dominant expression changes in CRC. In summary, these results show that Type 1 consists of tumors exhibiting a mesenchymal-like expression profile, while Type 2 contains epithelial tumors that are highly proliferative.

### Subtypes show selective pathway activation

SPEED identified several pathways to be activated in Subtype 1.1, including TGF-ß and VEGF, which are activated neither in 1.2 nor in 1.3 (Figure 3B). Furthermore, genes up-regulated in Subtype 1.1 were likely to be regulated by SRF (p-values < 1.15*10^-5^), which indicates activation of classical MAPK signaling in this subtype. Intriguingly, we found a significant up-regulation of the calcium signaling KEGG pathway (p-value = 0.01) in 1.1.

Strong activation of MAPK and JAK-STAT is unique to Subtype 1.2 while IL-1, MAPK+PI3K, TLR and TNF activation are significantly activated in both Subtype 1.1 and 1.2 (Figure 3B). With MSigDB, we identified significant overlap with targets of interferon regulatory factor 2 and 7 (IRF2, IRF7, p-value < 2.64*10^-2^), which agrees with the up-regulation of transcripts annotated with the GO terms ‘response to virus’, ‘response to other organism’, and ‘response to biotic stimulus’ (p-values < 8.8*10^-3^).

In Subtype 1.3, we identified genes annotated with several Gene Ontology (GO) [32] terms related to transport across membranes (p-values<0.05) to be up-regulated (Table 2).

SPEED identified a number of pathways to be activated in 2.1, while Subtype 2.2 showed no SPEED pathway activation (Figure 3C). Genes that are highly expressed in Subtype 2.1 are frequently annotated with the GO term “MAP kinase kinase kinase activity” (p-value = 0.02), confirming the activation of MAPK signaling in Subtype 2.1 found using SPEED. Intriguingly, we identified a number of genes to be up-regulated in Subtype 2.2 from two cytogenetic bands on the q-arm of Chromosome 20 (20q11 and 20q13, p-values < 5.68*10^-5^), and several bands on Chromosome 13q (13q13-14, 13q32-34, p-values < 3.96*10^-2^).

**References**

1. Devarajan K: **Nonnegative matrix factorization: an analytical and interpretive tool in computational biology.** *PLoS Comput Biol* 2008, **4**:e1000029.

2. Brunet J-P, Tamayo P, Golub TR, Mesirov JP: **Metagenes and molecular pattern discovery using matrix factorization.** *Proc Natl Acad Sci U S A* 2004, **101**:4164–4169.

3. Gaujoux R, Seoighe C: **A flexible R package for nonnegative matrix factorization.** *BMC Bioinformatics* 2010, **11**:367.

4. Yilmaz M, Christofori G: **EMT, the cytoskeleton, and cancer cell invasion.** *Cancer Metastasis Rev* 2009, **28**:15–33.

5. Gotzmann J, Fischer ANM, Zojer M, Mikula M, Proell V, Huber H, Jechlinger M, Waerner T, Weith A, Beug H, Mikulits W: **A crucial function of PDGF in TGF-beta-mediated cancer progression of hepatocytes.** *Oncogene* 2006, **25**:3170–3185.

6. Makagiansar IT, Williams S, Dahlin-Huppe K, Fukushi J, Mustelin T, Stallcup WB: **Phosphorylation of NG2 proteoglycan by protein kinase C-alpha regulates polarized membrane distribution and cell motility.** *J Biol Chem* 2004, **279**:55262–55270.

7. Efimova T, Broome A-M, Eckert RL: **A regulatory role for p38 delta MAPK in keratinocyte differentiation. Evidence for p38 delta-ERK1/2 complex formation.** *J Biol Chem* 2003, **278**:34277–34285.

8. Kurokawa M, Mitani K, Irie K, Matsuyama T, Takahashi T, Chiba S, Yazaki Y, Matsumoto K, Hirai H: **The oncoprotein Evi-1 represses TGF-beta signalling by inhibiting Smad3.** *Nature* 1998, **394**:92–96.

9. Kurokawa M, Mitani K, Yamagata T, Takahashi T, Izutsu K, Ogawa S, Moriguchi T, Nishida E, Yazaki Y, Hirai H: **The evi-1 oncoprotein inhibits c-Jun N-terminal kinase and prevents stress-induced cell death.** *EMBO J* 2000, **19**:2958–2968.

10. Liu Y, Chen L, Ko TC, Fields AP, Thompson EA: **Evi1 is a survival factor which conveys resistance to both TGFbeta- and taxol-mediated cell death via PI3K/AKT.** *Oncogene* 2006, **25**:3565–3575.

11. Khambata-Ford S, Garrett CR, Meropol NJ, Basik M, Harbison CT, Wu S, Wong TW, Huang X, Takimoto CH, Godwin AK, Tan BR, Krishnamurthi SS, Burris HA, Poplin EA, Hidalgo M, Baselga J, Clark EA, Mauro DJ: **Expression of epiregulin and amphiregulin and K-ras mutation status predict disease control in metastatic colorectal cancer patients treated with cetuximab.** *J Clin Oncol* 2007, **25**:3230–3237.

12. Li X-D, Miao S-Y, Wang G-L, Yang L, Shu Y-Q, Yin Y-M: **Amphiregulin and epiregulin expression in colorectal carcinoma and the correlation with clinicopathological characteristics.** *Onkologie* 2010, **33**:353–358.

13. Hering H, Sheng M: **Direct interaction of Frizzled-1, -2, -4, and -7 with PDZ domains of PSD-95.** *FEBS Lett* 2002, **521**:185–189.

14. Fearon ER: **Molecular genetics of colorectal cancer.** *Annu Rev Pathol* 2011, **6**:479–507.

15. Yang L, Lin C, Liu Z-R: **P68 RNA helicase mediates PDGF-induced epithelial mesenchymal transition by displacing Axin from beta-catenin.** *Cell* 2006, **127**:139–155.

16. Gandhirajan RK, Staib PA, Minke K, Gehrke I, Plickert G, Schlösser A, Schmitt EK, Hallek M, Kreuzer K-A: **Small molecule inhibitors of Wnt/beta-catenin/lef-1 signaling induces apoptosis in chronic lymphocytic leukemia cells in vitro and in vivo.** *Neoplasia* 2010, **12**:326–335.

17. Nawshad A, Lagamba D, Polad A, Hay ED: **Transforming growth factor-beta signaling during epithelial-mesenchymal transformation: implications for embryogenesis and tumor metastasis.** *Cells Tissues Organs* 2005, **179**:11–23.

18. Olie RA, Simões-Wüst AP, Baumann B, Leech SH, Fabbro D, Stahel RA, Zangemeister-Wittke U: **A novel antisense oligonucleotide targeting survivin expression induces apoptosis and sensitizes lung cancer cells to chemotherapy.** *Cancer Res* 2000, **60**:2805–2809.

19. Brabletz T, Jung A, Spaderna S, Hlubek F, Kirchner T: **Opinion: migrating cancer stem cells - an integrated concept of malignant tumour progression.** *Nat Rev Cancer* 2005, **5**:744–749.

20. Clark AB, Cook ME, Tran HT, Gordenin DA, Resnick MA, Kunkel TA: **Functional analysis of human MutSalpha and MutSbeta complexes in yeast.** *Nucleic Acids Res* 1999, **27**:736–742.

21. Yang Q, Zhang R, Wang XW, Linke SP, Sengupta S, Hickson ID, Pedrazzi G, Perrera C, Stagljar I, Littman SJ, Modrich P, Harris CC: **The mismatch DNA repair heterodimer, hMSH2/6, regulates BLM helicase.** *Oncogene* 2004, **23**:3749–3756.

22. van Engeland M, Derks S, Smits KM, Meijer GA, Herman JG: **Colorectal cancer epigenetics: complex simplicity.** *J Clin Oncol* 2011, **29**:1382–1391.

23. Liu W, Dong X, Mai M, Seelan RS, Taniguchi K, Krishnadath KK, Halling KC, Cunningham JM, Boardman LA, Qian C, Christensen E, Schmidt SS, Roche PC, Smith DI, Thibodeau SN: **Mutations in AXIN2 cause colorectal cancer with defective mismatch repair by activating beta-catenin/TCF signalling.** *Nat Genet* 2000, **26**:146–147.

24. Parikh JR, Klinger B, Xia Y, Marto JA, Blüthgen N: **Discovering causal signaling pathways through gene-expression patterns.** *Nucleic Acids Res* 2010, **38 Suppl**:W109–W117.

25. Subramanian A, Tamayo P, Mootha VK, Mukherjee S, Ebert BL, Gillette MA, Paulovich A, Pomeroy SL, Golub TR, Lander ES, Mesirov JP: **Gene set enrichment analysis: a knowledge-based approach for interpreting genome-wide expression profiles.** *Proc Natl Acad Sci U S A* 2005, **102**:15545–15550.

26. Kanehisa M, Goto S, Sato Y, Furumichi M, Tanabe M: **KEGG for integration and interpretation of large-scale molecular data sets.** *Nucleic Acids Res* 2012, **40**:D109–D114.

27. Schaefer CF, Anthony K, Krupa S, Buchoff J, Day M, Hannay T, Buetow KH: **PID: the Pathway Interaction Database.** *Nucleic Acids Res* 2009, **37**:D674–D679.

28. Ramírez F, Lawyer G, Albrecht M: **Novel search method for the discovery of functional relationships.** *Bioinformatics* 2012, **28**:269–276.

29. Grünert S, Jechlinger M, Beug H: **Diverse cellular and molecular mechanisms contribute to epithelial plasticity and metastasis.** *Nat Rev Mol Cell Biol* 2003, **4**:657–665.

30. Jechlinger M, Grunert S, Tamir IH, Janda E, Lüdemann S, Waerner T, Seither P, Weith A, Beug H, Kraut N: **Expression profiling of epithelial plasticity in tumor progression.** *Oncogene* 2003, **22**:7155–7169.

31. Loboda A, Nebozhyn MV, Watters JW, Buser CA, Shaw PM, Huang PS, Veer LV, Tollenaar RAEM, Jackson DB, Agrawal D, Dai H, Yeatman TJ: **EMT is the dominant program in human colon cancer.** *BMC Med Genomics* 2011, **4**:9.

32. Ashburner M, Ball CA, Blake JA, Botstein D, Butler H, Cherry JM, Davis AP, Dolinski K, Dwight SS, Eppig JT, Harris MA, Hill DP, Issel-Tarver L, Kasarskis A, Lewis S, Matese JC, Richardson JE, Ringwald M, Rubin GM, Sherlock G: **Gene Ontology: tool for the unification of biology. The Gene Ontology Consortium.** *Nat Genet* 2000, **25**:25–29.

Table S1: List of used CRC tumor sample expression datasets obtained from the Gene Expression Omnibus (GEO).

| GEO accession number | Number of samples | Reference |
| --- | --- | --- |
| GSE2109 | 293 (CRC samples) | Provided by the Expression Project for Oncology of the International Genomics Consortium (https://expo.intgen.org/geo/home.do) |
| GSE4107 | 22 | Hong et al., Clin Cancer Res, 2007, 13(4):1107-14 |
| GSE4183 | 53 | Gyorffy et al., PLoS One 2009, 4(5):e5645. |
| GSE8671 | 64 | Sabates-Bellver et al., Mol Cancer Res, 2007, 5(12):1263-75 |
| GSE10961 | 18 | Pantaleo et al., Br J Cancer, 2008, 99(10):1729-34 |
| GSE13067 | 74 | Jorissen et al., Clin Cancer Res, 2008, 14(24):8061-9 |
| GSE13294 | 155 | Jorissen et al., Clin Cancer Res, 2008, 14(24):8061-9 |
| GSE14333 | 290 | Jorissen et al., Clin Cancer Res, 2009, 15(24):7642-7651 |
| GSE15960 | 18 | Galamb et al, Br J Cancer, 2010, 102(4):765-73 |
| GSE17536 | 177 | Smith et al., Gastroenterology, 2010, 138(3):958-68 |
| GSE17537 | 55 | Smith et al., Gastroenterology, 2010, 138(3):958-68 |
| GSE20916 | 145 | Skrzypczak et al., PLoS One, 2010, 5(10) |
| GSE23878 | 59 | --- |
| GSE33113 | 90 | de Sousa et al., Clin Cancer Res, 2012, 18(11):3132-3141 |
| GSE37892 | 130 |  |

Table S2: List of publicly available cell line expression datasets. We included only CRC cell lines in our analysis.

| Identifier | Number of samples (CRC) | Reference/Link |
| --- | --- | --- |
| GSE8332 | 55 | Wagner et al., Nat Med, 2007, 13(9):1070-1077 |
| GSK | 60 | Greshock et al. Cancer Res. 2010 May 1;70(9):3677-86 <ftp://caftpd.nci.nih.gov/pub/caARRAY/transcript_profiling/> CRC lines in this set were originally sourced from ATCC |
| Sanger | 35 | Garnett et al., Nature, 2012, 483(7391):570-575 |

Table S3: Clinical staging of samples used for survival analysis.

| AJCC stage | | | |
| --- | --- | --- | --- |
| 1 | 2 | 3 | 4 |
| 28 | 145 | 133 | 56 |
| Duke’s stage | | | |
| A | B | C | D |
| 24 | 57 | 45 | 0 |

Table S4: Number of AZTS samples contained in core clusters and subtypes identified by iNMF.

| iNMF step 1 | Type 1 | | | Type 2 | |
| --- | --- | --- | --- | --- | --- |
| No. of samples in core cluster | 11 | | | 20 | |
| No. of samples in Type | 28 | | | 34 | |
| iNMF step 2 | Subtype 1.1 | Subtype 1.2 | Subtype 1.3 | Subtype 2.1 | Subtype 2.2 |
| No. of samples in core cluster | 4 | 4 | 7 | 7 | 11 |
| No. of samples in Subtype | 12 | 9 | 7 | 14 | 20 |

Table S13: Assignment of the combined cell line panel (AZCL, GSK, and GSE8332) to identified CRC subtypes

| Subtype 1.1 | Subtype 1.2 | Subtype 1.3 | Subtype 2.1 | Subtype 2.2 |
| --- | --- | --- | --- | --- |
| Colo320, NCIH716, SW480, SW620 | C10, C32, CaCo2, CaR1, CC20, CL11, Colo678, Colo741, HCT116, MDST8, NCI747, SW1417, SW480, SW837 | C170, DLD1, HCA7, HCT15, HCT8, LoVo, RKO, SNUC2B, SW48, Vaco5 | C125PM, CL34, Colo201, Colo205, Colo206, CX1, HT29, KM12, LS174T, LS180, LS411, LS513, NCIH548, SKCO1, WIDR | C106, C75, C80, C84, C99, CCK81, CL40, GP2d, HCA46, HRA19, HT55, LS1034, NCIH508, NCIH630, PC/JW, RCM1, SW1116, SW1222, SW1463, SW403, SW948, T84, Vaco10MS, Vaco4A, Vaco4S |

Table S14: Assignment of the Sanger cell line panel to identified CRC subtypes

| Subtype 1.1 | Subtype 1.2 | Subtype 1.3 | Subtype 2.1 | Subtype 2.2 |
| --- | --- | --- | --- | --- |
| C2BBe1, Colo320, Colo678, Colo741, NCIH716, SW620 | CaR1, HCT116, HCT15, LS123, NCIH747, RKO, SW48 | SW1417, SW837 | Colo205, HCC2998, HT29, KM12, LoVo, LS411N, RCM-1, SNUC2B, SW1116 | CW-2, GP5d, HT55, LS1034, LS513, NCIH630, SNUC1, SW1463, SW948, T84 |

Table S16: Compounds used for comparing pharmacological response of cell lines from the Sanger dataset assigned to different subtypes.

| Target | Compounds |
| --- | --- |
| SRC | Dasatinib, A-770041, WH-4-023, Bosutinib, AZD-0530 |


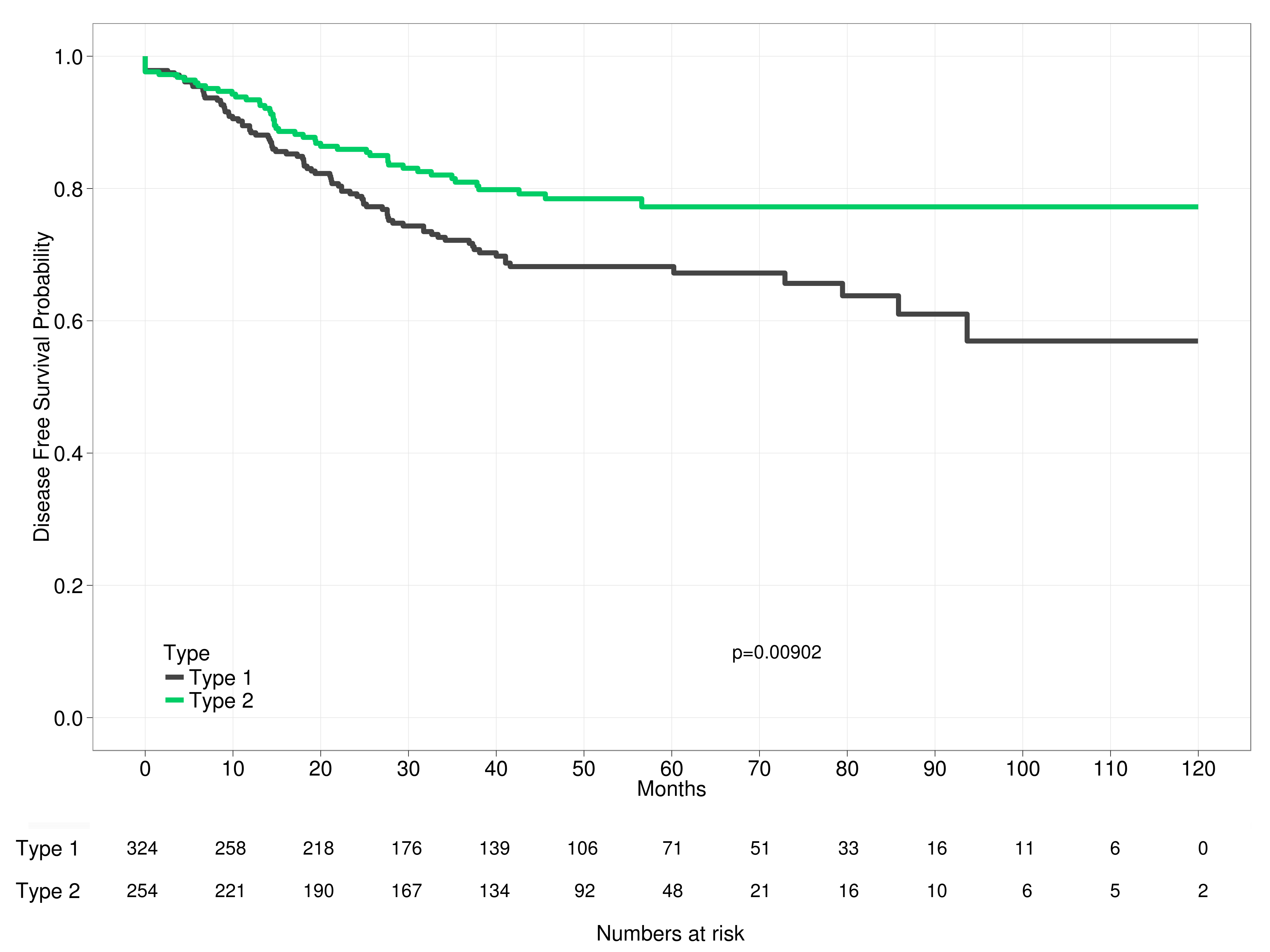
Figure S1: Difference in disease free survival between Types 1 and 2. The number of patients at risk is indicated above the x-axis at each time point (upper row for cluster 1, lower row for cluster 2). The plots include samples from GSE17536, GSE17537, GSE14333, GSE33113, and GSE37892.


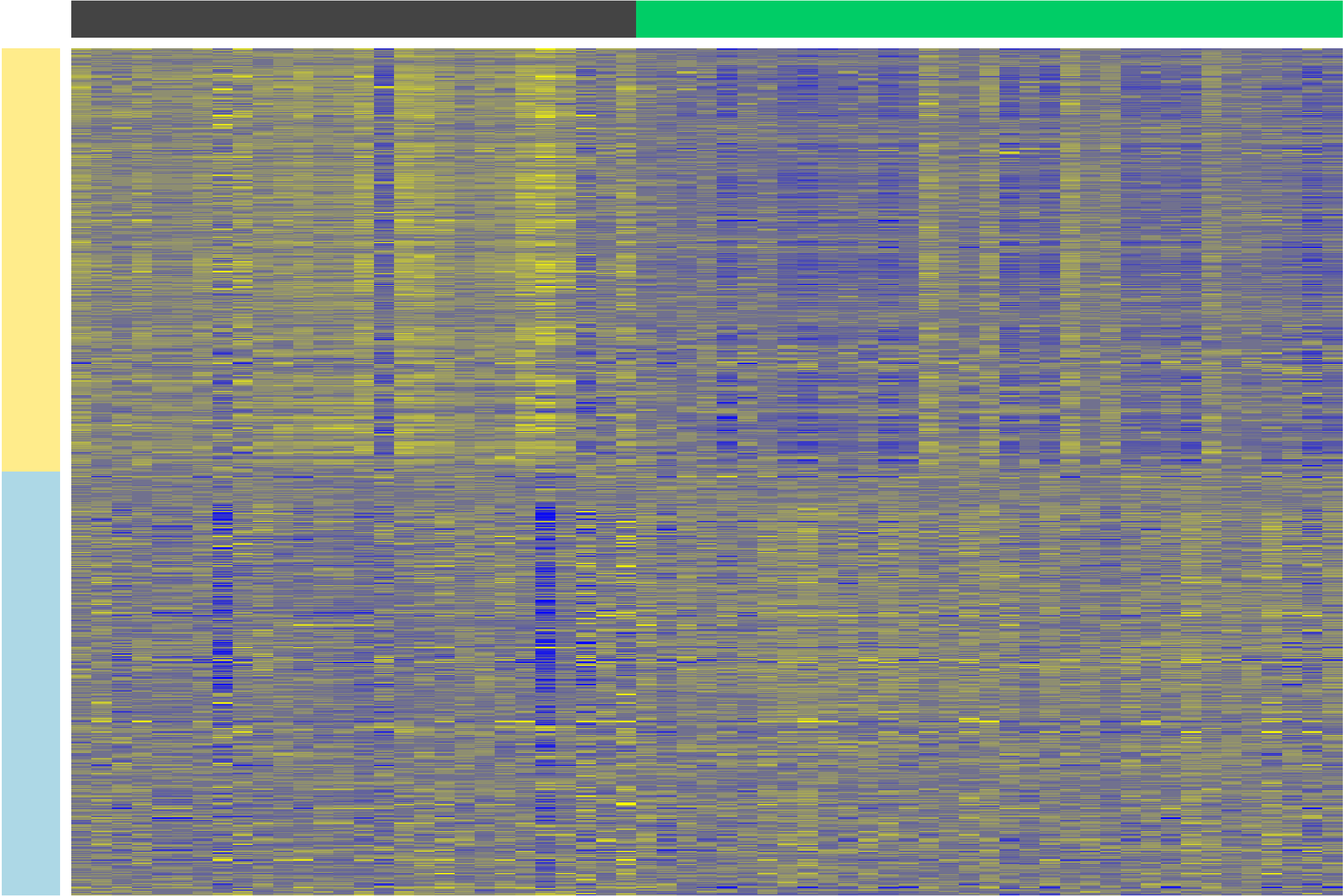


Figure S2: Expression pattern of EMT signature genes in Types 1 (black) and 2 (green). Mesenchymal marker genes (yellow bar) are highly expressed in Type 1 samples and repressed in epithelial Type 2 samples. The difference in expression of epithelial marker genes (light blue bar) is not as pronounced but tends to be higher in Type 2 samples.


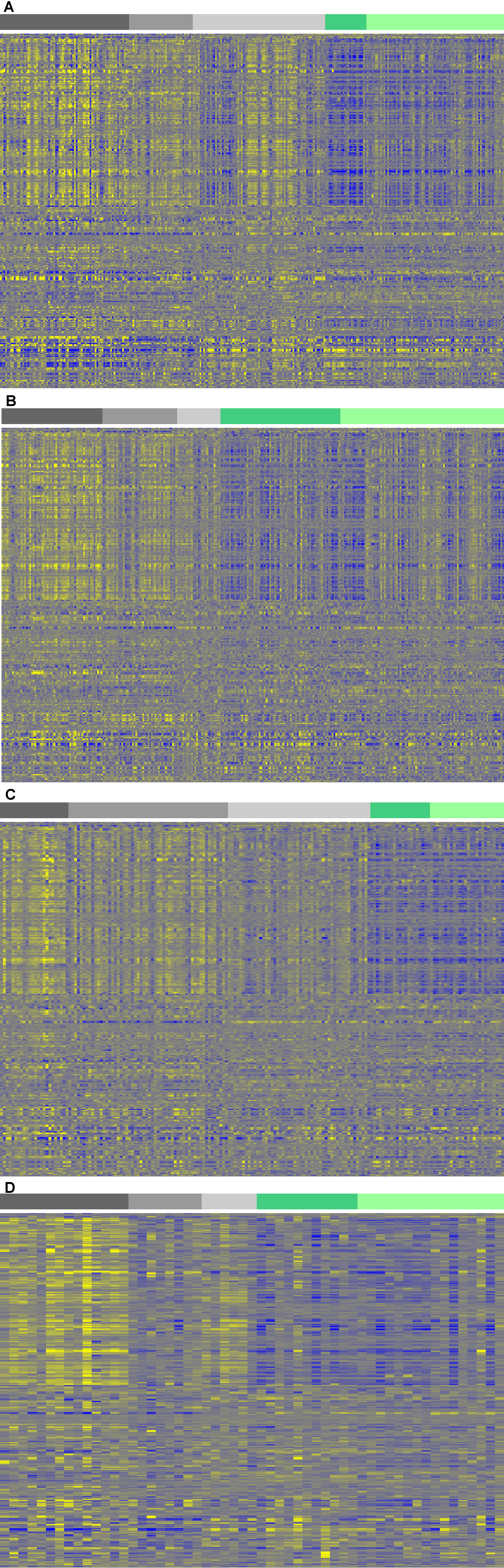
Figure S3: Expression of genes in the stromal signature identified by Perez-Villamil *et al*. in (A) GSE2109, (B) GSE14333, (C) GSE17536, and (D) GSE17537. Colored bars above the heatmaps show iNMF subtypes: black (1.1), dark gray (1.2), light gray (1.3), dark green (2.1), and light green (2.2). Expression values are shown in a color gradient ranging from blue indicating low expression to yellow indication high expression.


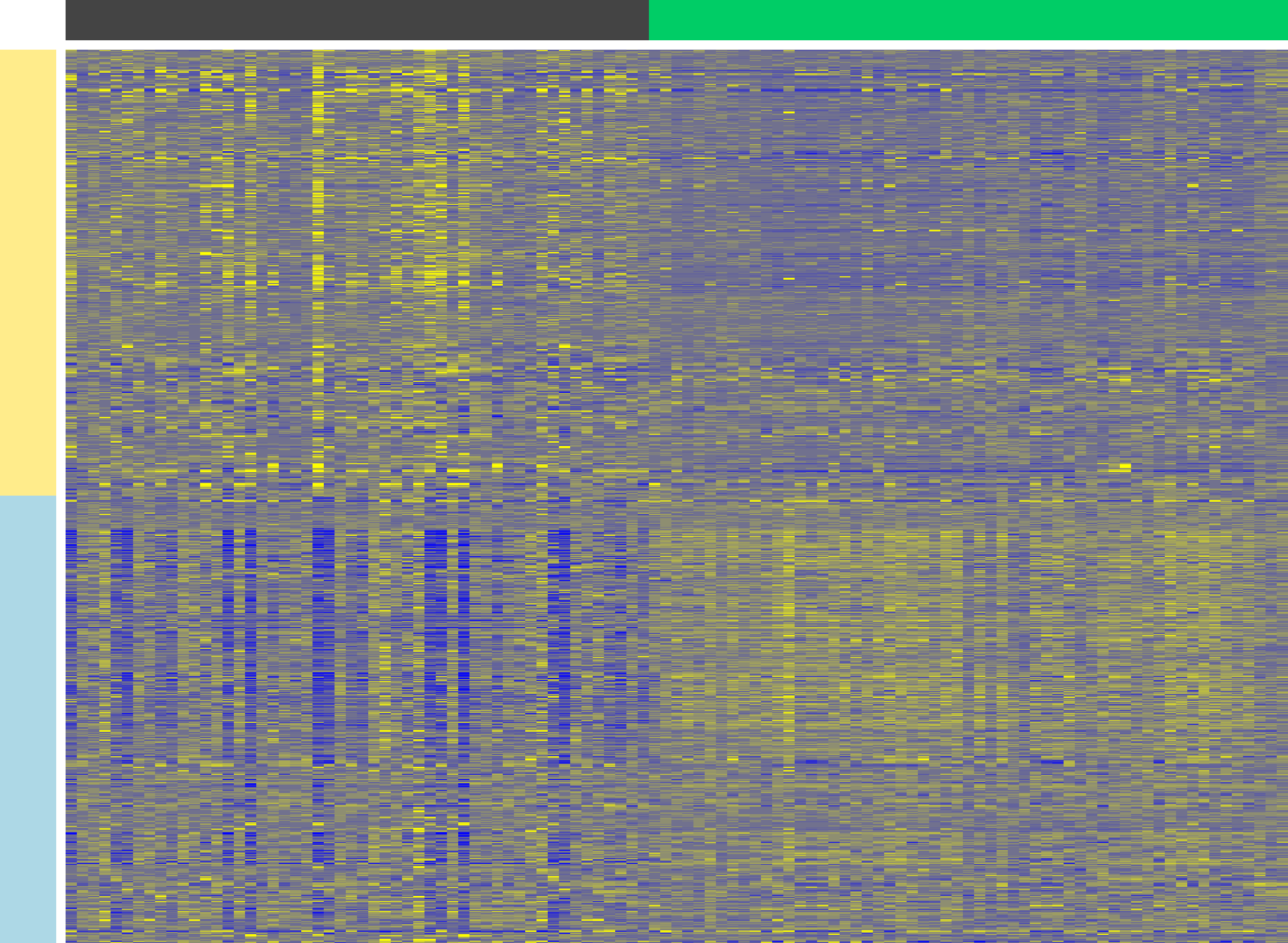
Figure S4: Expression pattern of EMT signature genes in cell lines assigned to Types 1 (black) and 2 (green). Mesenchymal marker genes (yellow bar) are highly expressed in Type 1 cell lines and repressed in epithelial Type 2 lines. The difference in expression of epithelial marker genes (light blue bar) is not as pronounced but tends to be higher in Type 2 lines.
