# Supplementary material for: Subtypes of primary colorectal tumors correlate with response to targeted treatment in colorectal cell lines
Source: BMC Med Genomics. 2012 Dec 31;5:66. doi: 10.1186/1755-8794-5-66 (PMC3543849; doi:10.1186/1755-8794-5-66)
Supplement: Additional file 9 — Table S12. Results of stratifying different colorectal cancer datasets. [file 1755-8794-5-66-S9.pdf]

Table S12: Results of stratifying different colorectal cancer datasets. Each table cell contains the number of samples from the respective dataset (column) in a particular cluster (row). The colored bars indicate the percentage of samples of a given dataset that have been assigned to the corresponding cluster. AZ TS denotes the tumor sample datasets used as input for iNMF, and GSK the GlaxoSmithKline cell line dataset, and Sanger the cell line panel from the Cancer Genome Project at the Wellcome Trust Sanger Institute .

	**AZ TS**	**GSE2109**	**GSE4107**	**GSE4183**	**GSE8671**	**GSE10961**	**GSE13067**	**GSE13294**	**GSE14333**	**GSE15960**	**GSE17536**	**GSE17537**
**Cluster 1**	28	189	7	27	32	9	34	83	126	6	130	28
**Cluster 2**	35	104	15	26	32	9	40	72	164	12	47	27
**Cluster 1.1**	12	75	5	13	21	5	12	17	58	2	24	14
**Cluster 1.2**	9	37	1	2	5	3	15	36	43	2	56	8
**Cluster 1.3**	7	77	1	12	6	1	7	30	25	2	50	6
**Cluster 2.1**	15	24	12	16	12	2	21	37	69	6	21	11
**Cluster 2.2**	20	80	3	10	20	7	19	35	95	6	26	16

	**GSE20916**	**GSE23878**	**GSE33113**	**GSE37892**
**Cluster 1**	105	24	36	80
**Cluster 2**	40	35	54	50
**Cluster 1.1**	24	10	5	16
**Cluster 1.2**	24	4	20	38
**Cluster 1.3**	57	10	11	26
**Cluster 2.1**	20	4	20	23
**Cluster 2.2**	20	31	34	27

	**AZ CL**	**GSK**	**GSE8332**	**Sanger**
	24	36	32	15
	31	24	23	19
	3	6	15	6
	16	24	11	7
	5	6	6	2
	22	6	9	9
	9	18	14	10
